# Can bradycardia pose as a “red herring” in neurosurgery? Surgical stress exposes an asymptomatic sick sinus syndrome: Diagnostic and management dilemmas

**DOI:** 10.4103/0972-5229.76088

**Published:** 2010

**Authors:** Ravi Dadlani, Koli Challam, Amit Garg, Alangar S. Hegde

**Affiliations:** **From:** Department of Pediatrics, Seth G.S. Medical College and KEM Hospital, Mumbai, India

**Keywords:** Bradycardia, Cushing’s reflex, posterior fossa surgery, sick sinus syndrome, sinus node dysfunction

## Abstract

Bradycardia in neurosurgery is almost always assumed to be secondary to intracranial conditions, specifically raised intracranial pressure causing Cushing’s reflex, the trigemino-cardiac reflex or brainstem lesions. We present a case of posterior fossa surgery in which persistent bradycardia developed in the postoperative period. A cardiac cause was initially overlooked since hydrocephalus was present preoperatively, which was initially assumed to be the cause of the bradycardia. The baseline pulse rate prior to surgery was 66 beats/minute. Only when repeated imaging revealed complete resolution of the hydrocephalus was a cardiology work up done and diagnosis of sick sinus syndrome established. The authors present an interesting case which demonstrates the need for a high degree of suspicion for such rare co-existing conditions. The diagnostic and management dilemmas are further discussed.

## Introduction

Bradycardia in neurosurgery is most often a component of the Cushing’s reflex (CR) triad of hypertension, bradycardia and apnea.[1] The list of perioperative conditions causing bradycardia in neurosurgical set-up is exhaustive.[1] Co-existing systemic or cardiac conditions that may be unmasked by surgical stress are rare associations.[2] Sick sinus syndrome (SSS) is a collection of clinical conditions characterized by sinus node dysfunction reflected as varied electrocardiographic abnormalities.[3] We report persistent bradycardia in the postoperative case of posterior fossa anaplastic ependymoma. The initial suspicion rested with neurological causes which were, however, ruled out by repeated clinical and radiological investigations. This report highlights the need for high vigilance in such an uncommon association and the need for a multidisciplinary approach in its diagnosis and management.

## Case Report

**Figure 1:**
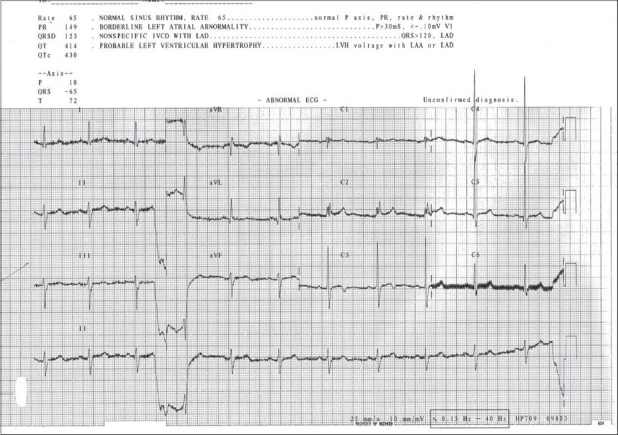
Preoperative Electrocardiography demonstrating sinus rhythm

**Figure 2:**
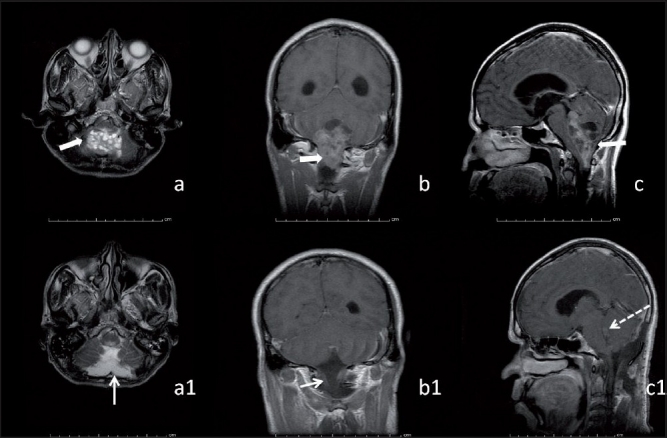
(a, b, c and a1, b1, c1): The preoperative (a, b, c) MRI of the brain compared with the postoperative MRI (a1, b1, c1). (a, a1) T2W axial images; (b, b1) axial T1W post-contrast enhancement images; (c, c1) post-contrast enhanced sag T1W images. The lesion was variegated in appearance, multicystic and revealed post-contrast enhancement. The posterior and lateral recesses of the fourth ventricle were displaced superiorly. The block arrows delineate the lesion in a–c. The thin white arrows in a1 and b1 demonstrate the postoperative cavity. The dashed arrow in c1 reveals a small residue at the floor of the fourth ventricle

A 40-year-old male presented with history of giddiness, gait ataxia and raised intracranial pressure (ICP) of 1 month duration. At presentation, his pulse rate was 66/minute and B/P was 110/70 mmHg. Electrocardiography (ECG) revealed sinus rhythm [Figure 1]. There was no history of any prior cardiac or other co-morbid conditions. Magnetic resonance imaging (MRI) of the brain revealed a midline posterior fossa lesion involving the dorsal brainstem, extending from the mid pons to the upper cervical spine [Figure 2a–c]. Anesthesia was induced with fentanyl 2 µg/kg and thiopentone 3 mg/kg. Muscle paralysis was achieved with pancuronium 0.1 mg/kg. Maintenance of anesthesia was carried with oxygen in nitrous oxide at a ratio of 50:50 and ventilated with volume controlled mode to mild hypocapneuic levels of pCO_2_ to 32 mmHg. The patient underwent a midline suboccipital craniotomy and decompression of the lesion. The tumor had a poor plane with the brainstem. Intraoperative bradycardia was recorded thrice. The first episode was related to tugging a small bit of tumor adherent to the floor of the fourth ventricle and the other two to placement of cottonoids, during hemostases, at the tumor bed. All three episodes ranged from a heart rate between 50 and 60 beats/minute, were transient (<1 minute) and recovered on discontinuing the offending stimulus.

**Figure 3:**
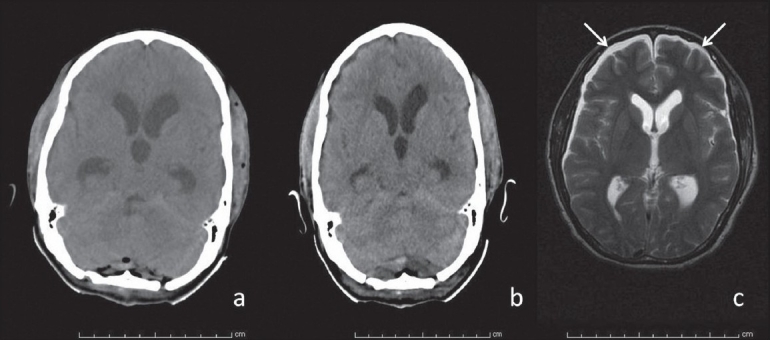
(a– c) Postoperative resolution of hydrocephalus: (a) 6 hours after surgery, (b) 24 hours later and (c) 36 hours after surgery. The bifrontal subdural hygromas are depicted by white arrows in c

Postoperatively, the patient was electively ventilated for 12 hours. A computed tomography (CT) scan of the brain [Figure 3a] done 6 hours post surgery revealed adequate decompression of the lesion but persisting hydrocephalus. A subsequent CT scan done 24 hours after surgery revealed a decrease in the size of the ventricles [Figure 3b].

**Figure 4:**
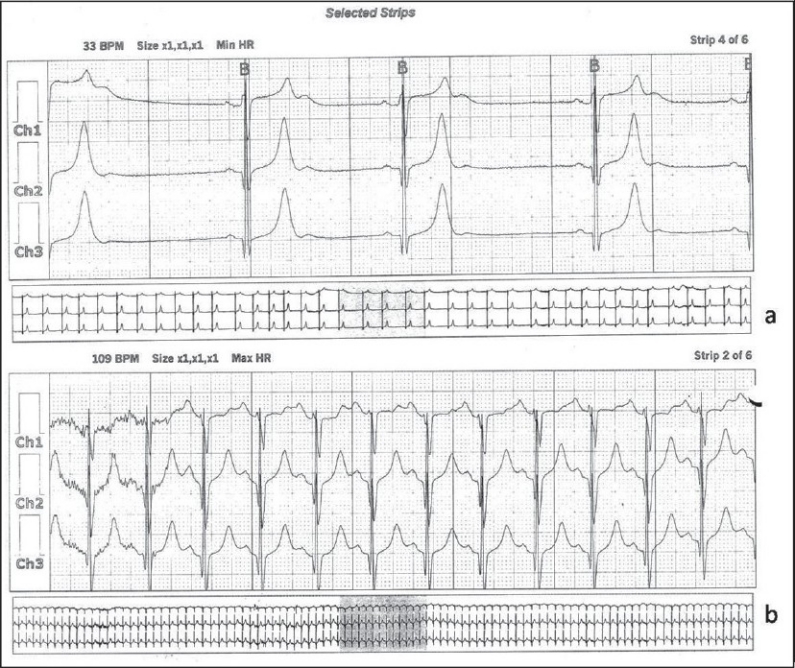
24-hour Holter recording demonstrating the typical bradycardia–tachycardia syndrome. Panel (a) reveals the bradycardia with a minimum pulse rate recorded as 33 beats/minute. (b) Delineates the tachycardia portion of the recording with a maximum pulse rate of 109 beats/minute

**Figure 5:**
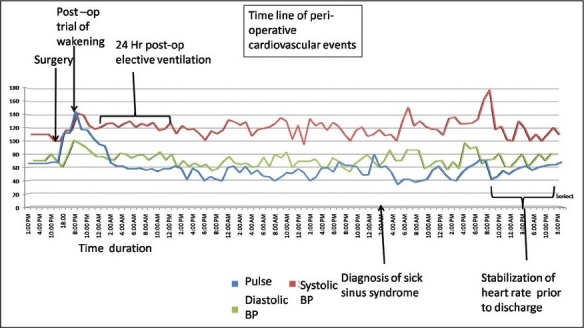
A graphical timeline of the important perioperative events recorded over a period of 10 days (see text for details)

The patient was electively ventilated and gradually weaned off during the first postoperative day. He demonstrated cardiovascular instability subsequent to weaning off with fluctuating pulse rate and predominant sinus bradycardia. The pulse rate at this point ranged from 50 to 65 beats/minute. The initial suspicion was related to the hydrocephalus and possible raised ICP. But during this time, the patient had been extubated, was conscious, alert and had no clinical features of raised intracranial tension. He developed a tachycardia-bradycardia syndrome on the second postoperative day. A formal cardiology evaluation was considered when an MRI of the brain [Figure 3c] revealed complete resolution of the hydrocephalus and thin bilateral subdural hygromas eliminating completely the possibility of hydrocephalus induced bradycardia. Initially, a 12-lead ECG and then a Holter recording [Figure 4] revealed SSS with the typical tachycardia-bradycardia rhythm with a variable heart rate from 33 to 109 beats/minute. The ECG revealed a 2:1 block during the bradycardia phase [Figure 4a and b]. In comparison to the preoperative ECG (sinus rhythm), the typical bradycardia-tachycardia syndrome and the 2:1 block were typical of the SSS. This patient responded well to initial doses of Atropine and subsequently was stabilized on Orciprenaline (Alupent^®^, German Remedies Mumbai, India) 10 mg t.i.d. Cardiac pacing was not deemed essential according to the AHA guidelines.[3] His heart rate stabilized between 65 and 70 beats/minute at discharge. A timeline graph of the perioperative cardiovascular events has been delineated in Figure 5.

## Discussion

Bradycardia in neurosurgery is associated with various conditions such as space occupying lesions (tumors, hematomas, etc.) to seizures, trigemino-cardiac reflex, cerebellar lesions, spinal lesions (neurogenic shock and autonomic dysreflexia) and Ondines curse.[1] The classical Cushing’s response to raised ICP in which the autonomic response ensures an adequate cerebral perfusion pressure despite an elevated ICP is responsible for most cases of postoperative bradycardia and may be a portent of impending herniation[4]. It may not be associated with the fully developed CR.[5] Bradycardia occurring from lesions arising from intrinsic brainstem lesions, especially at the ponto-medullary junction, is usually associated with other significant morbid symptomatologies[6,7] which were absent in this patient.

The management of associated hydrocephalus, as in this patient, is varied.[8] Complete resolution of hydrocephalus is reported in a majority of cases after adequate tumor decompression.[8]

SSS is a set of clinical conditions defined by sinus node dysfunction on ECG.[3] Most common etiology is idiopathic.[3] Most people with SSS are asymptomatic but features of cerebral hypoperfusion secondary to decreased cardiac output as a result of the arrhythmias may present with syncope or pre-syncope,[3] which was absent in this patient. Other symptoms may involve the cardiovascular system or have other systemic manifestations.[3] The ECG may vary from atrial bradyarrhythmias, atrial tachyarrhythmias, and alternating bradyarrhythmias and tachyarrhythmias. Holter recording is the commonest mode of diagnosing SSS.[3] Obviously, other described methods such as exercise testing, Valsalva’s maneuvers or carotid massage were unacceptable in this postoperative patient.[3]

### 

#### Anesthetic implications

Vagomimetic drugs such as Fentanyl, Propofol and Vecuronium should be used with caution for induction and maintenance of anesthesia in suspected cases.[9] There are a few case reports where asymptomatic SSS became overt after induction of general anesthesia.[9] Provision for percutaneous pacing should be made prior to general anesthesia. A recommendation for pacemaker implantation prior to general anesthesia even in an asymptomatic patient has been made in view of cardiovascular instability which may be induced by anesthesia.[10]

The recommended preoperative work up for a patient undergoing neurosurgery has included a general assessment to rule out diabetes mellitus, hypertension, renovascular diseases, carotid insufficiency and other systemic diseases. Specifically for posterior fossa surgery, assessment of lower cranial nerves and gag reflex, cervical spine diseases and right to left shunt has to be made, especially if the surgery is to be performed in the sitting position.[11] ECG abnormalities should be ruled out with a 12-lead ECG and an echocardiogram should be done. A high degree of suspicion should be maintained if the ECG should show any of the several arrhythmias known to be associated with SSS, especially since the patient may be clinically asymptomatic.[3]

## Conclusions

The purpose of this article is to highlight the possibilities of varied causes of co-existing bradycardia in a neurosurgical patient and to make the neurosurgical community aware of it. SSS, in its myriad forms, remains an under-diagnosed condition. A very high degree of suspicion is essential for the diagnosis of SSS since the patient would benefit from early medical management and possible external cardiac pacing in selected cases.
